# Therapeutic effects of *Lucilia sericata* larval excretion/secretion products on *Leishmania major* under in vitro and in vivo conditions

**DOI:** 10.1186/s13071-022-05322-7

**Published:** 2022-06-16

**Authors:** Jila Sherafati, Mohammad Saaid Dayer, Fatemeh Ghaffarifar

**Affiliations:** Department of Parasitology and Medical Entomology, Faculty of Medical Sciences, Tarbiat Modares University, Jalal AleAhmad Highway, Nasr, P.O. Box 14115-111, Tehran, Islamic Republic of Iran

**Keywords:** *Leishmania major*, Antileishmanial agents, Larval excretion, Larval secretion, *Lucilia sericata*, Maggot therapy, Cutaneous leishmaniasis

## Abstract

**Background:**

Leishmaniasis is a neglected infectious disease caused by protozoa of the genus *Leishmania*. The disease generally manifests as characteristic skin lesions which require lengthy treatment with antimonial drugs that are often associated with adverse side effects. Therefore, a number of studies have focused on natural compounds as promising drugs for its treatment. This study aimed to evaluate the effects of larval excretion/secretion products (ES) of *Lucilia sericata* in crude and fractionated forms on *Leishmania major*, by using in vitro and in vivo models.

**Methods:**

The in vitro experiments involved evaluation of ES on both promastigotes and macrophage-engulfed amastigotes, whereas the in vivo experiments included comparative treatments of skin lesions in *L. major*-infected mice with Eucerin-formulated ES and Glucantime.

**Results:**

The half maximal inhibitory concentrations of the crude ES, > 10-kDa ES fraction, < 10-kDa ES fraction, and Glucantime were 38.7 μg/ml, 47.6 μg/ml, 63.3 μg/ml, and 29.1 μg/ml, respectively. Significant differences were observed between percentage viabilities of promastigotes treated with the crude ES and its fractions compared with the negative control (*P* < 0.0001). The crude ES was more effective on amastigotes than the two ES fractions at 300 μg/ml. The macroscopic measurements revealed that the reduction of lesion size in mice treated with the crude ES followed quicker cascades of healing than that of mice treated with Glucantime and the ES fractions.

**Conclusions:**

The present study showed that the larval ES of *L. sericata* in both crude and fractionated forms are effective for both intracellular and extracellular forms of *L. major*. Also, the ES exert both topical and systemic effects on mice experimentally infected with *L. major*.

**Graphical abstract:**

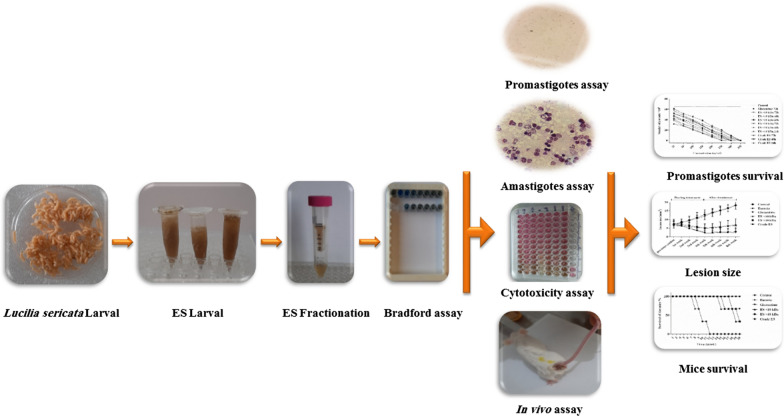

## Background

Leishmaniasis is a neglected tropical disease caused by protozoa of the genus *Leishmania* (Trypanosomatida: Trypanosomatidae) [1, 2]. It is estimated that approximately 350 million people who live in areas endemic for leishmaniasis are at risk of various forms of this disease [3]. About 20 species of *Leishmania* are known to be capable of infecting humans and a range of other animals. In humans, the parasite causes one of three clinical forms: visceral leishmaniasis (VL), cutaneous leishmaniasis (CL), and mucosal CL (MCL) [4]. The disease is mainly transmitted by bites of infected female sand flies belonging to the genera *Phlebotomus* and *Lutzomyia*, which occur in the Old World and the New World, respectively [5].

CL is the most common form of the disease, and causes disfiguring skin lesions with lifelong scarring [6]. Globally, leishmaniasis is responsible for a great number of disability-adjusted life years and large economic losses each year [7]. In endemic areas, the abundance of sand fly vectors, the high cost of drugs, prolonged treatments, and drug resistance are among the most serious setbacks for the control of this disease [8, 9]. In addition to the side effects of medications, the post-treatment scars of CL may be stigmatizing, especially for children [10]. Since the 1940s, pentavalent antimonial compounds such as Glucantime and Pentostam have been used as first-line drugs for the treatment of leishmaniasis [11]. Pentamidine, amphotericin B and paromomycin constitute the next choice of drugs, though the first two are contraindicated in pregnancy [12]. In Iran, antileishmanial therapy is currently based on the use of Glucantime ampoules [13]. With prolonged use, antileishmanial drugs can lead to clinical complications such as cardiac arrhythmia and anemia, and even toxicity and renal failure [11, 14, 15]. Given these problematics, alternative therapies have recently been suggested for CL treatment. These include the use of insect-derived natural compounds such as maggot-derived products, which have shown to have wound-healing effects [16, 17].

Maggot debridement therapy (MDT) has been widely used in the treatment of chronic wounds. MDT has been successfully used to treat necrotizing fasciitis; perianal gangrene; surgical wounds; burns; and venous, arterial and diabetic foot ulcers [18, 19]. The maggots of *Lucilia sericata* (Diptera: Calliphoridae) are usually used for MDT [20, 21]. These maggots exert a combination of wound debridement and disinfection as well as accelerated wound healing by secreting various enzymes such as proteases and nucleases, antimicrobial peptides and small active molecules [17, 22, 23]. Since its emergence 20 years ago, MDT has been increasingly recognized as a promising alternative therapy for wound healing which not only results in efficient wound debridement but also reduces the risk of post-surgery infections [18, 24, 25]. MDT has received approval as a medical device in many countries, including the USA (US Food and Drug Administration, 2004, case number K033391) [26].

The larval excretion/secretion products (ES) of *L. sericata* exhibit antimicrobial activity against both gram-positive and gram-negative bacteria [25, 27], as well as against protozoan agents of CL [28, 29]. In the present study, the effects of the crude and fractionated ES of *L. sericata* were comparatively investigated against *Leishmania major* using both in vitro and in vivo models. Also, in an attempt to find an alternative treatment for CL ulcers, the ES was formulated and tested against skin lesions of *L. major*-infected mice.

## Methods

### Collection and rearing of *L. sericata*

Wild adults of *L. sericata* were collected using bottle traps baited with raw chicken wing and liver in suburban areas of Saqqez City, Kurdistan Province, Iran between May and July 2020. The adult flies were anaesthetized with cold shock and morphologically identified using morphological keys [30, 31]. The fly colony was reared in mesh cages (60 × 60 × 60 cm) at the insectarium of the Medical Entomology Department of Tarbiat Modares University under the following conditions: 25 ± 1 °C, 60 ± 5% relative humidity and 16:8 h light/dark cycles [29]. Milk powder and sugar water solution (1:1 ratio) were supplied to feed the adults. Egg harvesting was performed by placing 150- to 200-g pieces of fresh beef liver in the rearing cage for 24 h as the oviposition substrates [32].

### Larval ES preparation and sterilization

About 100 stage II and III larvae of *L. sericata* were collected from established colonies. The larvae were starved for 6 h before being washed with 0.5% sodium hypochlorite followed by 5% formaldehyde and finally rinsed twice with sterile saline solution in a 50-ml Falcon tube for 5 min [33]. Subsequently, 1 ml of saline solution was added to the confined larvae and the tube was covered with aluminum foil and incubated at 37 °C for 1 h [33]. The larval ES were then collected by pipetting and centrifuged at 4000 *g* for 10 min [34].

### Bradford assay for protein measurement

A Bradford assay kit was used as a quick and ready-to-use colorimetric method for measuring the total protein content of the ES. The amount of protein in the solution was measured using a standard curve based on a serial dilution of known bovine serum albumin concentrations (0, 31.25, 62.5, 125, 250, 500, 1000 μg/ml). The protein samples were assayed using 8-well plates (SPL, Korea). The plates were incubated at 25 °C for 10 min in a dark place. Subsequently, optical absorption was read using an ELISA reader (model 680; Bio-Rad, Munich, Germany) at a wavelength of 595 nm [35].

### Fractionation of larval ES

The separation of the > 10-kDa ES and < 10-kDa ES fractions was achieved by centrifugation at 7500 *g* for 40 min using an Amicon Ultra-4 Centrifugal Filter Unit. The isolated fractions and the crude ES were filtered through a 0.22-µm syringe filter for sterilization. The sterilized ES was tested on blood agar medium to ensure that it was free of bacterial contamination. The ES were kept at − 20 °C until use.

### Larval ES protein profile determined by sodium dodecyl sulfate–polyacrylamide gel electrophoresis

The protein patterns of the ES samples of *L. sericata* were analyzed by electrophoresis on a 1-mm-thick 12.5% Tris–glycine sodium dodecyl sulfate–polyacrylamide gel. To this end, constant-current electrophoresis was performed at 110 V using Mini-PROTEAN 3 (Bio-Rad). After electrophoresis, the gels were stained with a solution containing Coomassie brilliant blue G250 and methanol. The mass-separated protein profiles were visualized against a pre-stained protein ladder (PAGEmark, 786-418) to estimate the molecular weights of the protein fractions.

### *Leishmania major* culture conditions

*Leishmania major* strain MRHO/IR/75/ER was maintained by regular passage through BALB/c mice. The amastigotes were isolated from spleens of infected BALB/c mice and developed into promastigotes on Novy-MacNeal-Nicolle medium. The logarithmic phase promastigotes at 2 × 10^6^ cells/ml were inoculated into Roswell Park Memorial Institute 1640 medium (Gibco, USA) supplemented with 10% heat-inactivated fetal bovine serum (FBS; Gibco) and 100 μg/ml penicillin–streptomycin (Thermo Fisher Scientific, USA). The promastigotes were grown in 25-ml cell culture flasks (Jet Biofil) and incubated at 26 °C until they reached the desired growth phase.

### Cell line culture

The mouse macrophage cell line (J774A.1) was obtained from the Pasteur Institute of Iran (Tehran). The macrophages were cultured in Dulbecco’s modified Eagle medium (Gibco) supplemented with 10% heat-inactivated FBS (Gibco) and 100 μg/ml penicillin–streptomycin (Thermo Fisher Scientific) at 37 °C in a humidified 5% CO_2_ incubator. The macrophages were grown in cell culture flasks (Jet Biofil).

### Promastigote survival assay

Logarithmic-phase promastigotes of *L. major* were cultured in Roswell Park Memorial Institute (RPMI) culture medium supplemented with 20% FBS in 96-well plates (SPL) at a concentration of 1 × 10^6^ cells/ml. A serial dilution was prepared from the crude and fractionated ES at initial concentrations of 350 µg/ml using RPMI 1640 medium. The ES dilutions were used to treat promastigotes aliquoted into 96-well plates. The negative control consisted of promastigotes cultured in the same medium without any ES treatment. The test plates were incubated at 26 °C and done in triplicate. The effects of ES dilutions on the survival of promastigotes were assessed by determining the multiplication of the promastigotes after 24, 48 and 72 h of incubation using a hemocytometer (Neubauer chamber) [36].

### ES cytotoxicity to macrophages determined by 3-(4.5-dimethylthiazol-2-yl)-2,5-diphenyltetrazolium bromide assay

The macrophages (J774A.1 cell line) were cultured in Dulbecco’s modified Eagle medium containing 10% FBS at 37 °C under a 5% CO_2_ atmosphere [37]. The macrophages at a concentration of 10^5^ cells/ml were aliquoted into each well of a 96-well plate (SPL). The larval ES of *L. sericata* and Glucantime were then added to the plates at various concentrations [negative control (0), 25, 50, 100, 150, 200, 250, 300 and 350 μg/ml]. The loaded plates were incubated at 37 °C for 72 h before 20 μl 3-(4.5-dimethylthiazol-2-yl)-2,5-diphenyltetrazolium bromide was added to each well. The plates were again incubated at 37 °C for 5 h. Finally, the supernatant was drained and replaced by 100 μl of dimethyl sulfoxide per well. After 15 min, the absorbance was read at 570 nm using an ELISA reader (model 680; Bio-Rad). The selectivity was calculated using the following formula: selectivity index = 50% cell cytotoxicity/inhibitory concentration for 50% of the parasites (half maximal inhibitory concentration; IC_50_).

### Amastigote susceptibility to larval ES

Twelve-well plates (SPL) were seeded with macrophages at a concentration of 2 × 10^6 ^cells/ml after a sterile coverslip (cover glass) had been placed on the bottom of each well. The plates were incubated at 37 °C for 24 h to allow the cells to adhere to the coverslips. The macrophages were then infected with *L. major* promastigotes (stationary phase) at 10:1 parasite:macrophage ratio and further incubated at 37 °C for 24 h. Next, free promastigotes were washed out with phosphate buffered saline and the adhered infected macrophages were exposed to a series of concentrations of (i) crude ES (150–300 μg/ml), (ii) > 10-kDa ES (150–300 μg/ml), (iii) < 10-kDa ES (150–300 μg/ml), and (iv) Glucantime (50–100 μg/ml). The tested concentrations were determined based on IC_50_ values obtained in the earlier promastigote survival assay. The tests were performed in triplicate. The 5th group of plates, the negative control, received no treatment. After 72 h of incubation, the coverslips inside the wells were fixed with methanol, stained with 10% Giemsa and examined using light microscopy. The number of infected macrophages and the average number of parasites per macrophage were counted per 100 cells [36].

### Development of ulcers

Thirty female BALB/c mice (4–6 weeks old) were obtained from Razi Vaccine and Serum Research Institute (Karaj, Iran). The mice were divided into six groups, each consisting of five animals. Each group was kept in a separate cage in the stress-free animal house of Tarbiat Modares University and fed ad libitum. The stationary-phase promastigotes are more resistant, active and efficient cells. They are obtained when the parasite population ceases growth. This is determined by daily sampling and counting of promastigotes. The inocula were injected subcutaneously into mice at the base of the tail. Injections were performed by insulin syringe under aseptic conditions under a laminar air flow cabinet. CL lesion development was monitored on a weekly basis until ulceration in the 5th week post-inoculation, after which the treatments were applied. Lesions were measured before and after treatments and their size used as an indication of wound-healing effect. The weekly measurements of the lesions were continued for a further 4 weeks.

### ES preparations for the treatment of ulcers

To obtain formulated ointments to treat the lesions of the infected mice, Eucerin was added to each of the crude ES and their fractions at a ratio of 1:1. The concentration of the ES fractions used as treatments was 300 μg/ml. The ointments formulated with the crude ES, the ES fraction > 10 kDa and the ES fraction < 10 kDa were used to treat the 1st, 2nd and 3rd groups of infected mice, respectively. As a positive control, the 4th group was treated with subcutaneous injection of Glucantime around the lesion (60 mg/kg per day for 28 days) and the 5th group was left without treatment as a negative control. Finally, the 6th group was treated with pure Eucerin to evaluate its possible impact on lesion healing. All the materials were freshly prepared before application.

### Parasite load evaluation

Eight weeks after treatment of the lesions, two mice from each of the six groups were sacrificed to determine the number of live *L. major* parasites in their infected spleens using the parasite-limiting dilution assay. For this purpose, 30 mg of spleen tissue was used to prepare a serial dilution of 1–10^−15^ of the parasite before being cultured in a 96-well plate containing complete medium enriched with 20% FBS, and streptomycin at 100 μg/ml [38]. The plates were then incubated at 26 °C for 10–14 days. Three replications were performed for each piece of mouse spleen, i.e. six replicates per group. Finally, the total number of positive wells (presence of motile promastigotes) and negative wells (absence of motile promastigotes) was identified under an inverted light microscope [39].

The following equation [40] was used to determine the parasite burden:

$${\text{parasite}}\,{\text{burden}} = - \log \,\left( {{\text{parasite}}\,{\text{dilution/spleen}}\,{\text{weight}}} \right)$$

### Statistical analyses

The experimental results were analyzed by* t*-test and one-way ANOVA in GraphPad Prism version 6.07. To determine the independence of two categorical variables, χ^2^ and/or Fisher’s exact tests were also undertaken. The data are presented as means ± SD. Dose–response curves were drawn using non-linear regression. The area under the curve was used to estimate the larval ES effect against *L. major* promastigotes. The Bradford equation was applied through online software at (https://www.aatbio.com) and the graph was plotted using GraphPad Prism. Indices such as infection rate, decrease in infection rate, percentage viability, decrease in percentage viability, parasite load and survival index of amastigotes were determined using the equations presented in Rahimi et al. [41]. Differences were considered statistically significant at *P* < 0.05.

## Results

### Bradford assay

**Fig. 1:**
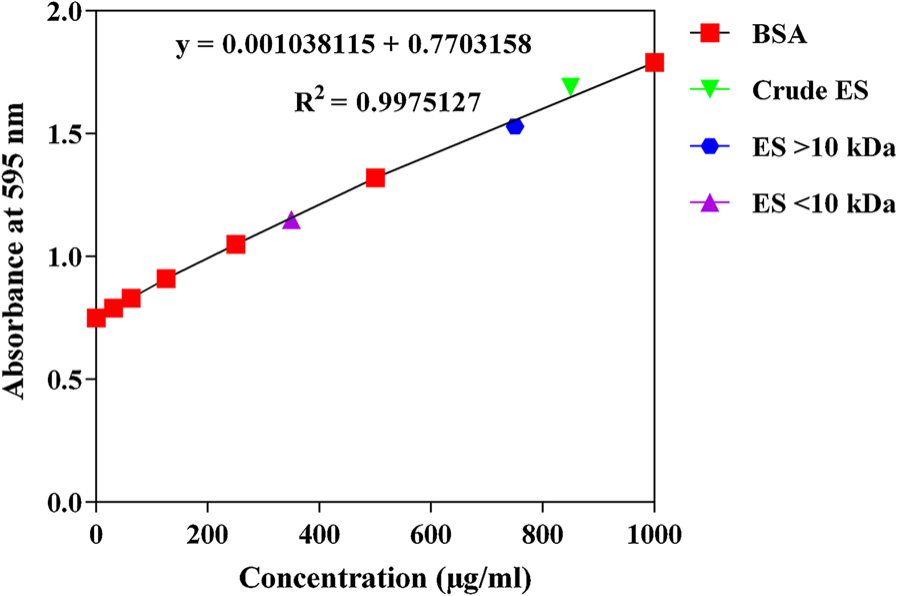
Bradford assay standard curve and the plotted excretion/secretion product (*ES*) sample concentrations.* BSA* Bovine serum albumin

A linear relationship was observed between protein concentration and absorbance at 595 nm (Fig. 1). This enabled the calculation of the corresponding linear equation and hence the protein concentrations of the ES samples. Given that the average net absorbance values at 595 nm for the crude ES, > 10-kDa ES and < 10-kDa ES were 1.69, 1.53 and 1.15, respectively, the average protein concentrations were 885.92, 731.80 and 366.71 μg/ml, respectively (Fig. 1). Therefore, the maximum protein concentration of ES samples used in this study was set at 350 μg/ml.

### Larval ES protein profiles

**Fig. 2:**
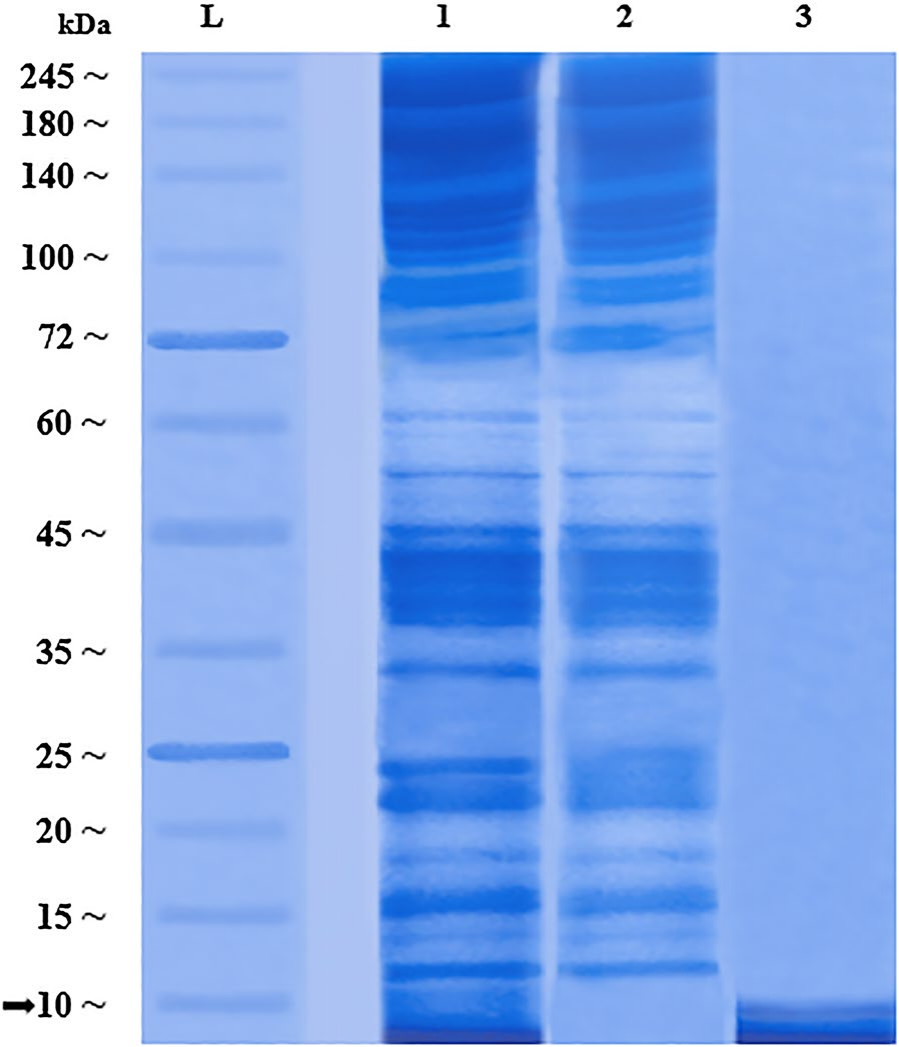
Sodium dodecyl sulfate–polyacrylamide gel electrophoresis patterns of ES protein profiles of *Lucilia sericata* larvae. *L* Pre-stained protein ladder (PAGEmark, 786–418), *lane 1* crude ES, *lane 2* > 10-kDa ES fraction, *lane 3* < 10-kDa ES fraction

The electrophoretic protein patterns of larval ES of *L. sericata* are shown in Fig. 2. The protein bands indicate clear fractionation at a 10-kDa cut-off, with no or minimal loss of ingredients. The relatively high recovery of the ES samples, which only required mild processing, indicated that the methodology used was reliable for the production of ES fractions for use in bioassays against leishmanial cells. About 20–25 protein bands were observed for the crude ES, which matched bands in both the > 10-kDa and < 10-kDa fraction. This shows that the precision of the fractionation of the crude ES had an accuracy of almost 100% with no or only minor effects on the resulting protein profiles.

### Sensitivity of promastigotes to ES

**Fig. 3:**
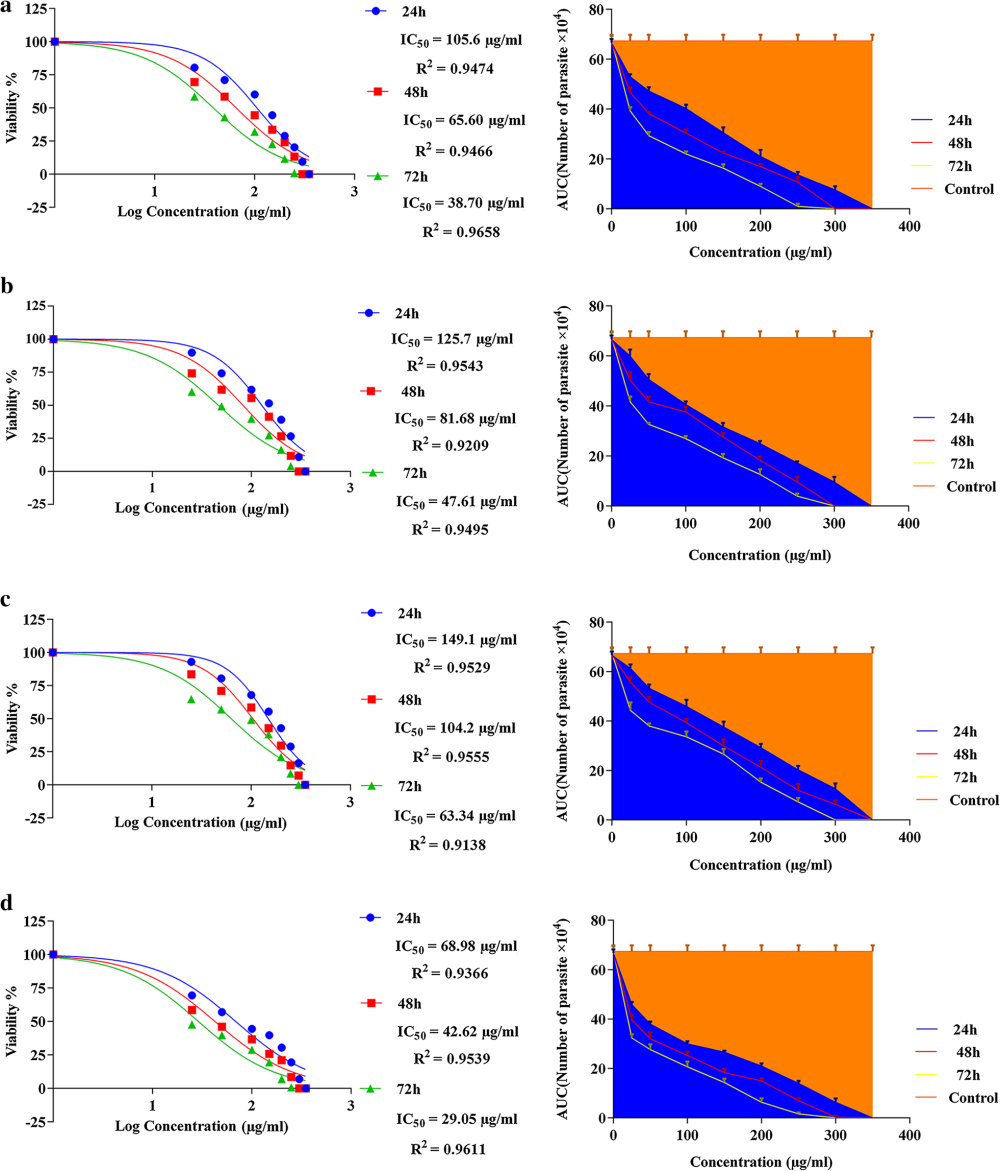
**a**–**d** Dose–response curves of tested ES of *Lucilia sericata* larvae and Glucantime against *Leishmania major* promastigotes (half maximal inhibitory concentration;* IC*_50_) at 24 h, 48 h and 72 h. Area under the curve indicates the relationship between the number of parasites and the tested doses compared with the negative control. **a** Crude ES, **b** > 10-kDa ES, **c** < 10-kDa ES, **d** Glucantime

**Fig. 4:**
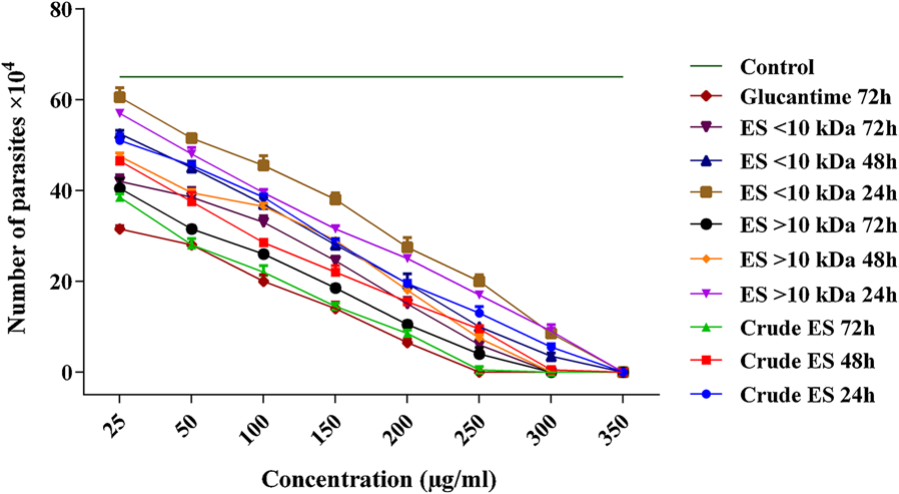
Number of *Leishmania major* promastigotes per milliliter upon exposure to different concentrations of the crude and fractionated ES compared with Glucantime at three time points (24, 48 and 72 h). Data are presented as means ± SD; means are significantly different at *P* < 0.05

The IC_50_ values of the larval ES of *L. sericata* against promastigotes were evaluated at 24, 48 and 72 h. The lowest IC_50_ values for the crude ES, > 10-kDa ES and < 10-kDa ES were 38.70 μg/ml (log = 1.588), 47.61 μg/ml (log = 1.667) and 63.34 μg/ml (log = 1.802), respectively, at 72 h. The results were compared with those of Glucantime (Fig. 3). The area under each curve shows the corresponding number of parasites as a function of the drug concentration. These results support the data presented in Fig. 4. The percentage viabilities of the promastigotes exposed to different doses of the crude ES, > 10-kDa ES and < 10-kDa ES were significantly different than that of the negative control (*P* ≤ 0.001). While < 10-kDa ES was significantly less effective than Glucantime with respect to reducing promastigote viability (*P* = 0.001), no significant differences were observed between the percentage viabilities of promastigotes exposed to the crude ES and the > 10-kDa ES and those exposed to Glucantime at 72 h post-exposure (*P* = 0.841 and *P* = 0.860, respectively). It was obvious that higher concentrations of the ES fractions at longer exposure times were more toxic for promastigotes. Therefore, the toxic effect of ES was considered to be both dose and exposure time dependent (Fig. 4).

### ES cytotoxicity to macrophages

**Fig. 5:**
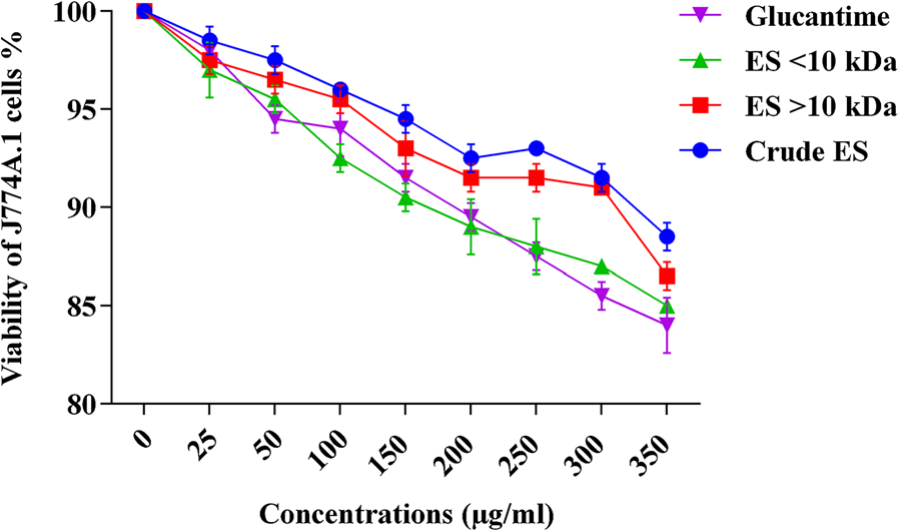
The number of viable J774A.1 cells treated with increasing doses of ES of *Lucilia sericata* larvae and Glucantime at 72 h of exposure. There were no significant differences between the treatments (*P* = 0.343)

Figure 5 shows the cytotoxicity of various concentrations of ES fractions and Glucantime to macrophage cell line J774A.1 after 72 h of exposure. The ES fractions exerted similar, non-significantly different, mild effects on the percentage viability of macrophages. However, the crude ES was as toxic as Glucantime to macrophages, and there was no significant difference between their cytotoxicity (*P* = 0.340). The selectivity indices for the crude ES, > 10-kDa ES and < 10-kDa ES were 3.87, 2.79 and 1.58, respectively.

### Amastigote susceptibility to larval ES fractions

**Fig. 6:**
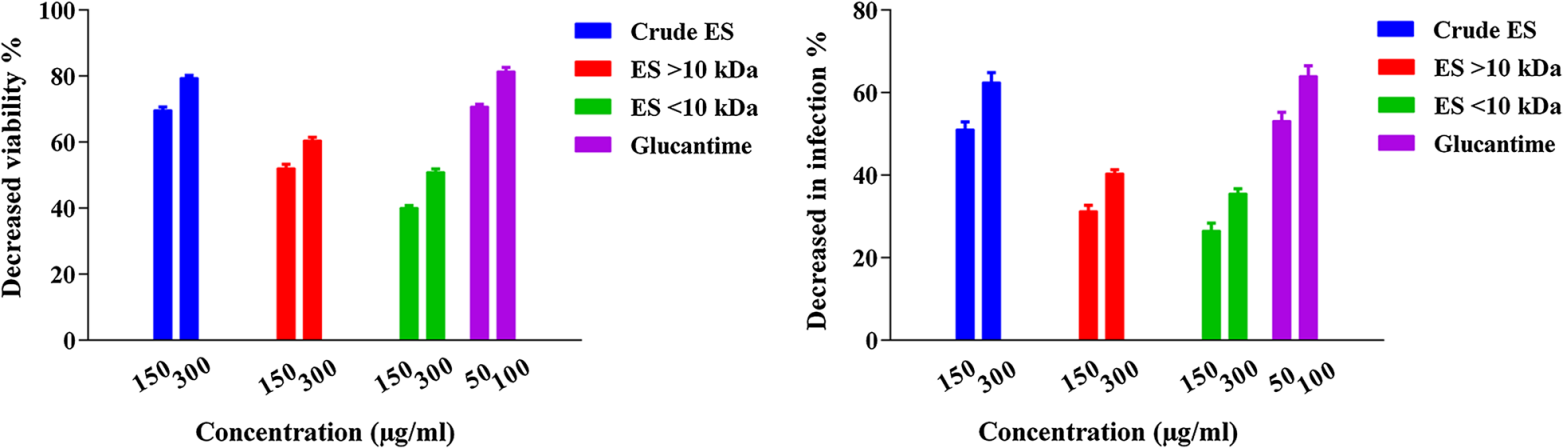
Reduction in infection rate and viability of amastigotes upon treatment with the crude ES, fractionated ES and Glucantime

Table 1 indicates that infection rates of macrophages and the percentages of viable amastigotes inside them were considerably reduced at 72 h of exposure to larval ES fractions when compared with the control group (*P* = 0.001 and *P* = 0.001, respectively). The macrophage infection rates were reduced to a similar level by > 10-kDa ES and < 10-kDa ES (*P* = 0.476), though these treatments were significantly less effective than Glucantime (*P* = 0.015 and *P* = 0.007, respectively). However, crude ES and Glucantime were similarly effective in reducing macrophage infection rates, with no significant difference between them (*P* = 0.749) (Fig. 6). In addition, no significant difference was observed between the effect of > 10-kDa ES and that of the < 10-kDa ES (*P* = 0.290) in reducing the mean number of amastigotes per infected macrophage. Also, both ES fractions were significantly less effective than Glucantime (*P* = 0.029 and *P* = 0.01, respectively) in reducing the number of amastigotes per macrophage. However, crude ES and Glucantime exerted similar effects (*P* = 0.789). Treatment with crude ES at higher concentrations reduced the infection rate of macrophages and the viability of amastigotes (*P* = 0.036). Treatment with crude ES at 300 μg/ml lead to a significant reduction in the parasite loads of macrophages, equal to 1.75 ± 0.05 per cell, compared with the negative control. Treatment with Glucantime at 50 μg/ml also reduced parasite loads, to 1.96 ± 0.07 amastigotes per macrophage. The survival index of amastigotes was lowest, i.e. 53.72 ± 2.44, upon treatment with crude ES at 300 μg/ml (Table 1).

**Table 1:** Parameters indicative of *Leishmania major* amastigote viability and infectivity to J774A.1 in the crude ES, fractionated larval ES and Glucantime treatments

Treatment	Dosage (μg/ml)	Infected cells (%)	Viability of amastigotes (%)	Parasite load	Survival index
Control	0	85.37 ± 3.58	100 ± 0.00	3.19 ± 0.01	270.9 ± 1.15
Crude ES	150	41.66 ± 1.28*	30.27 ± 1.93*	1.97 ± 0.02*	82.23 ± 2.35*
	300	32.00 ± 2.43*	20.58 ± 2.74*	1.75 ± 0.05*	53.72 ± 2.44*
> 10-kDa ES	150	58.33 ± 2.75	49.90 ± 2.71*	2.28 ± 0.03	132.99 ± 2.01*
	300	51.00 ± 1.55*	39.46 ± 3.42*	2.10 ± 0.01*	107.33 ± 2.51*
< 10-kDa ES	150	62.66 ± 2.38	59.93 ± 2.86*	2.60 ± 0.10	162.73 ± 2.10*
	300	55.00 ± 3.10*	49.02 ± 3.53*	2.42 ± 0.00*	133.21 ± 2.38*
Glucantime	50	39.66 ± 2.08*	28.54 ± 0.56*	1.96 ± 0.07*	77.67 ± 1.53*
	100	30.85 ± 1.53*	15.51 ± 1.12*	1.68 ± 0.02*	51.01 ± 2.01*

*P < 0.05 (significantly different from the negative control)

### Effect of larval ES fractions on leishmanial lesions

The skin lesions of all of the infected mice began with redness and swelling at the site of injection in the 3rd week post-inoculation. The swelling increased gradually, and lead to crust formation and the development of gangrene in the 4th week. The mean lesion measurements are presented in Table 2. The lesions of control mice increased progressively in size until the 4th week post-inoculation, where they reached a mean size of 12.8 ± 2.86 mm^2^. Likewise, the lesions increased in size in Eucerin-treated mice, and reached a mean size of 12 ± 1.87‌ mm^2^ over the same period. There was no statistically significant difference between the lesion sizes of control and Eucerin-treated mice (*P* = 0.782). This indicates that Eucerin has no therapeutic effect when applied on its own. In contrast, the lesions started to decrease gradually in size in mice treated with the crude ES. The mean lesion sizes were 2.6 ± 1.19 mm^2^ in the crude ES-treated group, 5 ± 2.35 mm^2^ in the > 10-kDa ES-treated group, 5.2 + 2.280 mm^2^ in the < 10-kDa ES-treated group and 2.2 ± 1.327 mm^2^ in the Glucantime-treated group. Although the crude ES and Glucantime were similarly effective in reducing the lesion sizes, with no significant difference between these treatments (*P* = 0.489), when lesion sizes in these treatments were compared with those in the > 10-kDa ES and < 10-kDa ES treatments, the differences were statistically significant (*P* < 0.001). In fact, the > 10-kDa ES and < 10-kDa ES treatments were similarly less effective against lesion development, and there was no significant difference between them with respect to reducing lesion sizes (*P* = 0.747) (Table 2).

**Table 2:** The effects of the crude ES and fractionated ES at 300 μg/ml on lesion size (mean ± SD) of *Leishmania*-infected mice versus positive and negative controls at different time points post-infection

Groups	Initial lesion size (mm^2^)	Post-treatment lesion size (mm^2^)			
		First week	Second week	Third week	Fourth week
Crude ES	7.6 ± 2.61	6.6 ± 2.50	4.8 ± 2.59*	3.8 ± 2.05*	2.6 ± 1.82*
> 10-kDa ES	7.4 ± 2.07	7.2 ± 1.92	6.4 ± 1.82	5.8 ± 1.92*	5 ± 2.35*
< 10-kDa ES	6.6 ± 2.05	6.4 ± 2.30	5.8 ± 2.77	5.4 ± 2.30*	5.2 ± 2.28*
Glucantime	6.8 ± 1.72	6.4 ± 2.15	4.8 ± 1.72*	3.6 ± 1.74*	2.2 ± 1.33*
Eucerin	7.4 ± 1.82	8.4 ± 1.80	9.4 ± 1.86	10.6 ± 1.72	12 ± 1.87
Control	7 ± 2	8.2 ± 1.92	9.6 ± 2.30	11.2 ± 2.77	12.8 ± 2.86

*P < 0.05 (significantly different from the negative control)

**Fig. 7:**
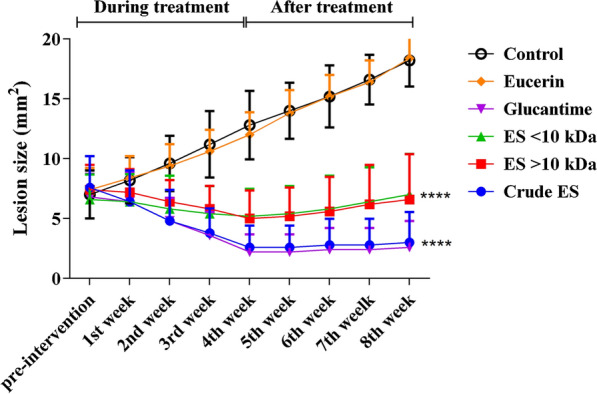
Progression of leishmanial lesion size in treated and control BALB/c mice from pre-intervention stages until the 8th week post-treatment (differences are considered statistically significant at *P* < 0.05; ****P < 0.001)

After the termination of the treatment period, the measurement of the lesions was continued for several weeks to assess the possibility of a recurrence of inflammation. A slight increase in lesion size was observed in the case of the crude ES-treated (3.0 ± 2.55 mm^2^) and Glucantime-treated (2.6 ± 1.96 mm^2^) groups. In contrast, the wounds erupted upon the cessation of treatment in mice treated with > 10-kDa ES (6.6 ± 3.78 mm^2^) and < 10-kDa ES (7 ± 3.39 mm^2^) (Fig. 7).

### Parasite loads

**Fig. 8:**
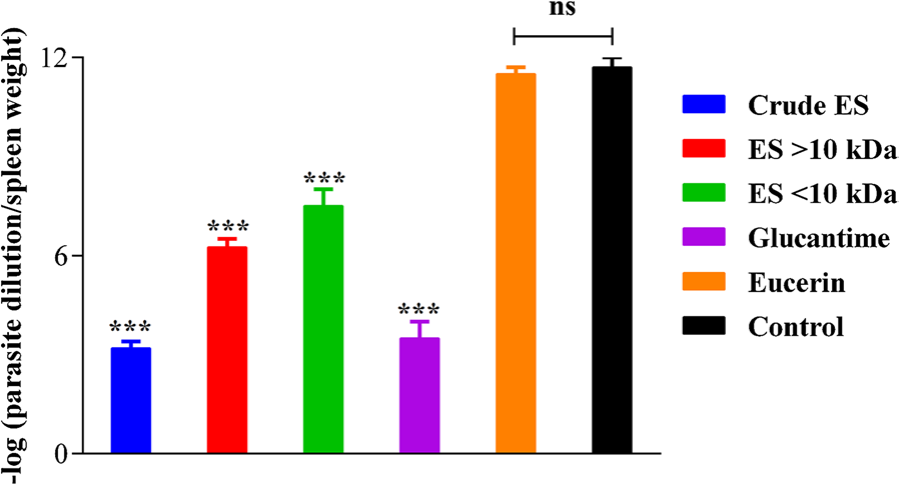
Parasitic loads of the spleens of mice infected with *Leishmania major* at 8th week post-treatment. Data are presented as means ± SD of triplicates (***P = 0.001,* ns* not significant)

The parasite loads of the spleens of mice infected with *L. major* were determined using a parasite-limiting dilution assay. The parasite load was significantly lower in treated groups compared with the negative control group (*P* < 0.001). There was no significant difference between the latter group and the Eucerin-treated groups in parasite loads (*P* > 0.05). The crude ES-treated group had the lowest parasite load when compared with all the treated groups (*P* < 0.001) with the exception of the Glucantime-treated group (*P* = 0.267). As seen in the other comparisons, there was no statistically significant difference between the parasite loads of the groups treated with > 10-kDa ES and < 10-kDa ES (*P* = 0.059) (Fig. 8).

### Mice mortality during the study phases

**Fig. 9:**
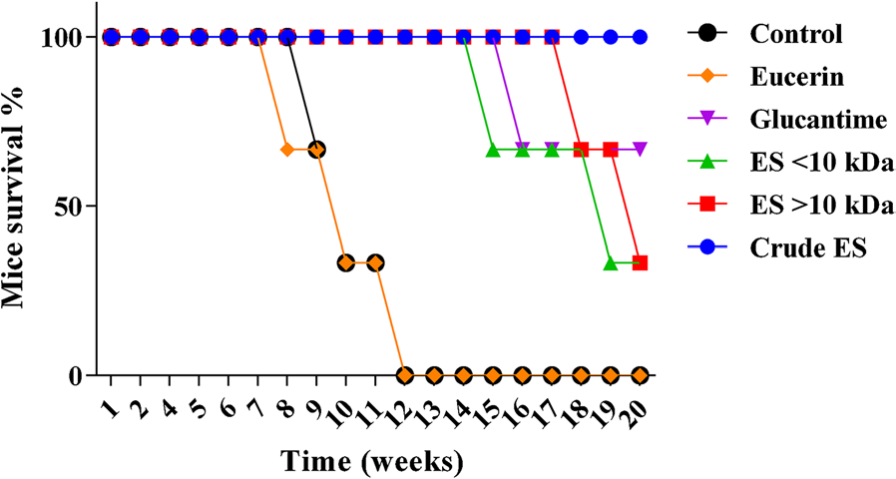
Survival of treated and untreated mice over a period of 20 weeks post-infection with *Leishmania major* promastigotes (differences are significant at *P* < 0.05)

The mortality of the experimental mice was monitored from the beginning of the treatments until 20 weeks post-treatment. Mortality in the negative control and Eucerin-treated mice occurred after the 7th week post-treatment and increased rapidly, so that all of the mice were dead by week 13 post-treatment. In contrast, mortality in the ES-treated mice did not change after week 14 post-treatment, so that more than 60% of these mice were still alive by the end of week 20. Mortality in the negative control and Eucerin-treated groups was similarly high, with no significant difference between them (*P* = 0.813). However, both of these groups differed significantly from the other treatment groups in their mortality rates (*P* < 0.001). The lowest mortality rates were observed in the crude ES-treated group and the Glucantime-treated group, with no significant difference between them (*P* = 0.239). By the end of week 20 post-inoculation, the survival rates of mice treated with > 10-kD ES and < 10-kDa ES were similar at 20%; although there was no significant difference between them (*P* = 0.346), they were significantly different from the survival rates of the crude ES and Glucantime-treated groups (*P* < 0.001) (Fig. 9).

## Discussion

Leishmaniasis is a major health problem in tropical and subtropical regions of the world [29, 42]. Pentavalent antimonial compounds remain the first-line therapies for leishmaniasis, but entail painful injections and a long course of administration, have side effects, and are costly [43, 44]. Therefore, research has been devoted to finding cheaper and more effective drugs for leishmaniasis, especially among natural products, with minimum or no side effects [43]. Many studies have examined the cytotoxic effects of larval ES of various species of flies, including *Lucilia sericata*, against different *Leishmania* species, both under in vitro and in vivo conditions [29, 45, 46]. In the present study we evaluated the anti-leishmanial activity of the crude and fractionated ES of *Lucilia sericata* against promastigotes and amastigotes of *Leishmania major*, both under in vitro and in vivo conditions, using BALB/c mice as an animal model. In this study we also examined the cytotoxic effects of the ES against *L. major* promastigotes and macrophage cell line J774A.1. To the best of the our knowledge, this is the first comparative study of the effects of *Lucilia sericata* crude ES and its fractions on *Leishmania major* and macrophages.

In this study, the highest rates of cytotoxicity of the highly concentrated ES on the macrophages were 15%, 13% and 12% for > 10-kDa ES, < 10-kDa ES and the crude ES, respectively (Fig. 4). These results contrast with those of a study by Sanei-Dehkordi et al. [29] in which the cytotoxicity of *L. sericata* larval ES to the same macrophage cell line was reported to be 40%, although ambiguity regarding the exact concentration of their applied ES makes comparison difficult. However, testing *L. sericata* hemolymph and saliva on the same cell line, Rahimi et al. [41] reported lower toxicity to macrophages at a level comparable to that recorded in our study. Evaluating the effect of the ES derived from *L. sericata* and *Sarconesiopsis magellanica* on a human lung cell line (MRC5), Laverde-Paz et al. [47] showed that it had no effect on cell survival rates at a low concentration (10 μg/ml), but was effective in reducing cell survival rates at a higher concentration (20 μg/ml). The toxicity of an ES seems to be a function of various factors including insect species, rearing methods, ES concentration and storage conditions, as well as the types of exposed cells.

In our study, all larval ES of *L. sericata* were effective against promastigotes, but to various degrees. The crude ES was more lethal than the > 10-kDa ES and < 10-kDa ES fractions. These findings are consistent with those of other studies in which the effects of ES, hemolymph, and saliva of *Lucilia sericata* larvae were evaluated against *Leishmania tropica* both under in vivo and in vitro conditions [28, 41]. Similar results have been reported by other authors examining promastigote susceptibility to larval ES [48, 49].

The antibiotic properties of *L. sericata*-derived ES have been shown against fungi as well as gram-positive and gram-negative bacteria [50, 51]. ES fractions of *L. sericata* with molecular weights of < 1 kDa and 3–10 kDa have been shown to exert antibacterial activity against gram-positive and gram-negative bacteria including *Pseudomonas aeruginosa*, *Klebsiella pneumoniae* and *Staphylococcus aureus* [52]. The results of the present study showed that the ES fraction with a molecular weight of < 10 kDa had slightly lower anti-leishmanial activity than the ES fraction of higher molecular weight (> 10 kDa). However, the crude ES showed the highest toxicity to *L. major*, both under in vitro and in vivo conditions. Therefore, for an effective and strong anti-leishmanial activity, apparently all ES constituents, of different molecular weights, are required. This argument is supported by the results of the sodium dodecyl sulfate–polyacrylamide gel electrophoresis, which showed that the fractionation process caused no loss of proteins according to the protein profiles of the resulting ES fractions (Fig. 2).

An analysis of the susceptibility of intracellular amastigotes of *Leishmania major* to the ES of *Lucilia sericata* showed that the parasites are more vulnerable to the ES at high concentrations than at low concentrations. The ES significantly reduced the parasite’s survival rate. This finding contrasts with data reported in two studies [29, 48] which used *Leishmania major* and *Leishmania panamensis* amastigotes to infect the macrophage cell line J774 and U937 cell line, respectively. The authors postulated that the applied ES were more toxic at low concentrations than at high concentrations. In the present study, the lowest percentage viabilities of amastigotes were 20.6 ± 2.7 and 15.5 ± 1.1, following application of the crude ES (300 μg/ml) and Glucantime (100 μg/ml), respectively (Table 1; Fig. 5). The survival index values upon treatment with the crude ES were lower than those obtained with > 10-kDa ES and < 10-kDa ES in amastigote-infected macrophages (J774A.1). Also, a considerable reduction in the survival index was seen in the ES-treated cells compared with the control cells (Table 1). It is noteworthy that the anti-leishmanial effects of the crude ES and its fractions may be controlled by adjusting their concentrations [41, 53]. In the present study, the parasite load and survival index were both determined under in vitro and in vivo conditions. In both cases, the crude ES and Glucantime induced the lowest parasite loads. Also, significant decreases in parasite load and survival index were observed in groups treated with ES compared with the negative control groups (Table 1; Fig. 8).

In this study, lesions of BALB/c mice infected with *L. major* showed a significant reduction in size upon treatment with the crude ES and Glucantime, averaging 5 mm^2^ and 4.6 mm^2^, respectively (Table 2). The lesions treated with the crude ES, > 10-kDa ES and < 10-kDa ES were significantly smaller than those left untreated or treated with Eucerin (Fig. 7). Using *Lucilia sericata* maggots to directly treat the lesions of BALB/c mice infected with *Leishmania major*, Kabiri et al. [46] did not find any significant difference between the treated and untreated lesions*.* This indicates that the extracted ES of *L. sericata* larvae is more effective than the debridement activity of the larvae in healing leishmanial wounds. A study by Sanei-Dehkordi et al. [29] confirmed that ES extracts of *Lucilia sericata* and *Calliphora vicina* larvae were highly effective in reducing the size of lesions of BALB/c mice infected with *Leishmania major* when compared with the negative control. A similar result [28] also confirmed the effectiveness of larval ES of *Lucilia sericata* in healing the leishmanial ulcers of BALB/c mice infected with *Leishmania tropica* compared with the control group (*P* < 0.001). However, another study [33] showed that maggot therapy and the ES derived from *Lucilia sericata* and *S. magellanica* larvae were similarly effective in treating lesions caused by *Leishmania panamensis* in hamsters. The efficacy of *L. sericata* larval ES in reducing the development of the leishmanial lesions was attributed to its potency in skewing the monocyte-macrophage differentiation from pre-inflammatory to pro-angiogenic pathways [54].

Various studies have shown the potential therapeutic effects, under both in vitro and in vivo conditions, of the larval ES of different fly species on different species of *Leishmania*, including *Leishmania amazonensis* [55], *Leishmania tropica* [28], *Leishmania major* [29, 46], and *Leishmania panamensis* [33]. Here, we also clearly showed the anti-leishmanial activity of the larval ES of *Lucilia* *sericata* on the intracellular and extracellular forms of the parasite *Leishmania major* both under in vitro and in vivo conditions. We also provide evidence that the ES of larval *L. sericata* has both topical and systemic therapeutic effects on leishmanial lesions of the model animal used here.

## Conclusions

To the best of our knowledge, this is the first report of the fractionation of the ES of larval *L. sericata* into two fractions of different molecular weight, > 10 kDa and < 10 kDa. Microscopic and macroscopic evaluation showed that both fractions are effective in the treatment of both intracellular and extracellular forms of the parasite *L. major*, although the > 10-kDa fraction was slightly more effective than the < 10-kDa fraction. However, the crude ES showed a higher antileishmanial activity than the fractionated ES. This study revealed that *Lucilia sericata* crude ES and its fractions are effective candidates for the treatment and cure of lesions induced by *Leishmania major*. However, the addition of suitable adjuvants may reinforce their effectiveness, and this deserves further study.

## Data Availability

The data supporting the conclusions of the present study are available from the corresponding author on reasonable request.

## Abbreviations

CL: Cutaneous leishmaniasis
ES: Excretion and secretion products
FBS: Fetal bovine serum
IC_50_: Half maximal inhibitory concentration
MDT: Maggot debridement therapy
RPMI: Roswell Park Memorial Institute
